# Linkage mapping and QTL analysis of flowering time in faba bean

**DOI:** 10.1038/s41598-021-92680-4

**Published:** 2021-07-02

**Authors:** David Aguilar-Benitez, Inés Casimiro-Soriguer, Fouad Maalouf, Ana M. Torres

**Affiliations:** 1 Área de Genómica y Biotecnología, IFAPA Centro “Alameda del Obispo”, Apdo 3092, 14080 Córdoba, Spain; 2 International Center for Agricultural Research in the Dry Areas (ICARDA), Beirut, Lebanon

**Keywords:** Agricultural genetics, Genetic markers, Plant breeding, Plant genetics

## Abstract

Flowering time marks the transition from vegetative to reproductive growth and is key for optimal yield in any crop. The molecular mechanisms controlling this trait have been extensively studied in model plants such as *Arabidopsis thaliana* and rice. While knowledge on the molecular regulation of this trait is rapidly increasing in sequenced galegoid legume crops, understanding in faba bean remains limited. Here we exploited translational genomics from model legume crops to identify and fine map QTLs linked to flowering time in faba bean. Among the 31 candidate genes relevant for flowering control in *A. thaliana* and *Cicer arietinum* assayed, 25 could be mapped in a segregating faba bean RIL population. While most of the genes showed conserved synteny among related legume species, none of them co-localized with the 9 significant QTL regions identified. The *FT* gene, previously implicated in the control of flowering time in numerous members of the temperate legume clade, mapped close to the most relevant stable and conserved QTL in chromosome V. Interestingly, QTL analysis suggests an important role of epigenetic modifications in faba bean flowering control. The new QTLs and candidate genes assayed here provide a robust framework for further genetic studies and will contribute to the elucidation of the molecular mechanisms controlling this trait.

## Introduction

With its global production of 4.92 million metric tons^1^ grown in 2.51 million ha, faba bean (*Vicia faba* L.) is the fourth most widely grown cool season legume after pea (*Pisum sativum*), chickpea (*Cicer arietinum*) and lentil (*Lens culinaris*). Due to its valuable content of protein and energy, faba bean is used as a livestock feed in both developing and developed countries, and as staple food in developing regions, especially in North and East Africa. Moreover, faba bean contributes to sustainable agriculture and plays an important role in the management of soil fertility through crop rotation and nitrogen fixation. Nevertheless, environmental conditions together with pests, diseases and weeds constrain the production of this major food crop.

Faba bean plants are particularly susceptible to frost, high temperature and/or low moisture during floral development and anthesis^2^. Therefore, the time of flowering is a key trait in faba bean breeding in order to minimize exposure to critical stress and to produce novel varieties that are better adapted to local environments^3^. While two environmental factors, photoperiod and temperature, play a critical regulatory role in plant flowering, the response to these factors varies considerably among genotypes. Faba beans accessions are classified as, in general, day-neutral or long-day adapted, but photoperiod-unresponsive genotypes have also been identified^4–8^.

Flowering time has been studied for decades both in model and crop plants^9^. A long list of regulatory genes have been identified in *A. thaliana* whose orthologues have also been identified in legume crops such as *Glycine max*^10^ or *Pisum sativum*^11^. Despite some examples of gene loss or duplication, the main flowering genes are well catalogued and largely conserved among legumes^10,12–14^.

**Figure 1:**
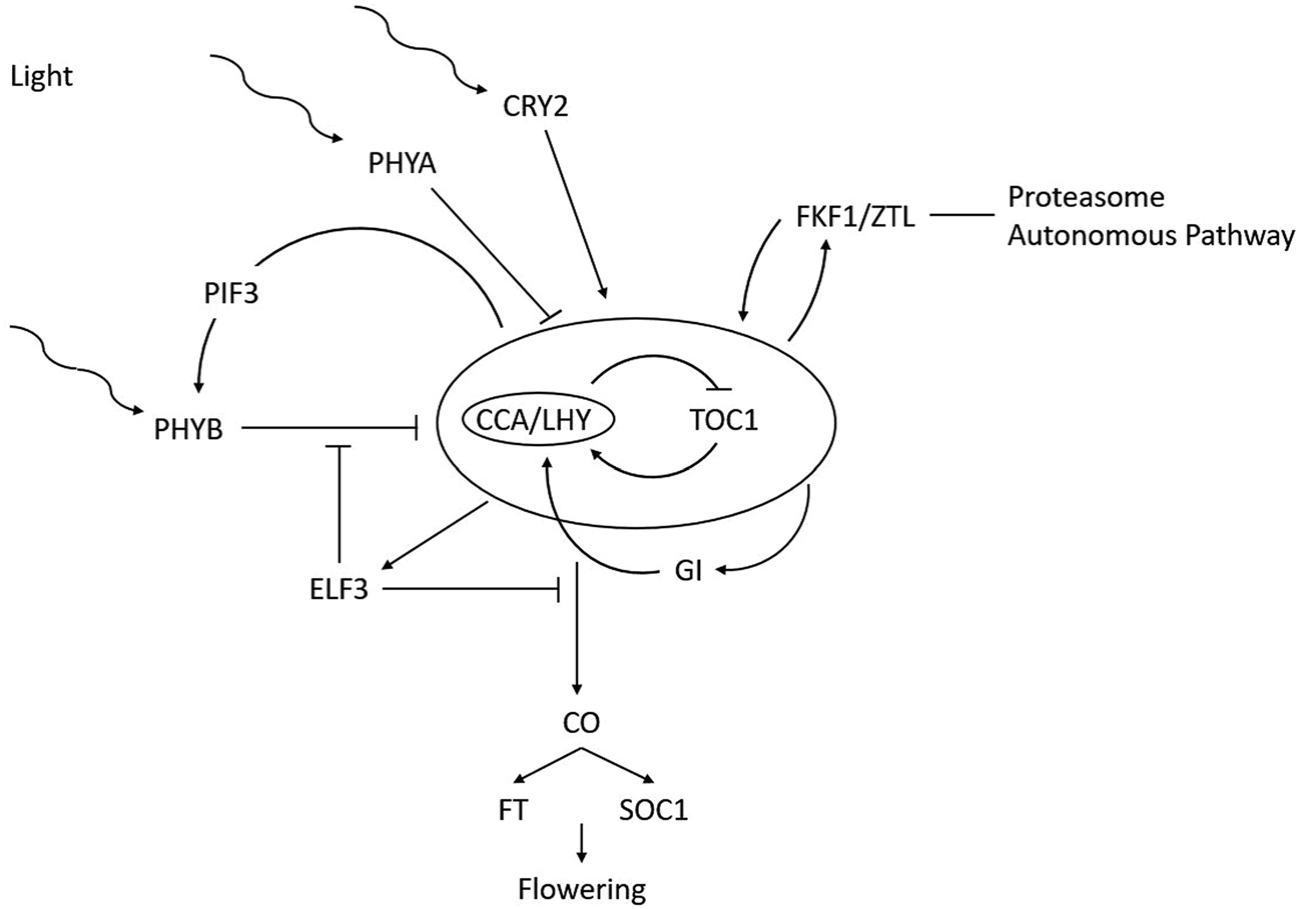
Schematic model of the genetic pathway of flowering time control by the photoperiod. The central circle represents the circadian clock. Modified from Mouradov et al. (2002).

Floral initiation depends on a system where photoreceptors perceive changes in daylength (photoperiod) and trigger the plant responses. Shim^15^ divided the photoperiodic flowering mechanisms into three steps: light input, circadian clock and output. Light information is integrated into innate photoperiodic timing mechanisms governed by the circadian clock to induce genes that trigger flowering. In legumes different classes of photoreceptors have been identified: three phytochromes (*PHYA*, *PHYB* and *PHYE*) and two cryptochromes (*CRY1* and *CRY2*)^12,16^. In addition to the light signals, internal pathways convey information via the FKF1*/ZTL* gene family^17^. All these signals converge on the circadian clock, which is controlled by the transcription factors *CIRCADIAN CLOCK ASSOCIATED 1* (*CCA1*) and *LATE ELONGATED HYPOCOTYL* (*LHY*), *TIMING OF CAB 1* (*TOC1*), *EARLY FLOWERING 3* (*ELF3*) and *GIGANTEA* (*GI*). In long day (LD) plants, such as *Arabidopsis* or galegoid legumes, light signals promote the expression of *CONSTANS* (*CO*), which activates *FLOWERING LOCUS T* (*FT*) and *SUPPRESSOR OF OVEREXPRESSION OF CO1* (*SOC1*) to promote flowering at the meristem^18^ (Fig. 1). FT genes have an important position within the genetic hierarchy that controls flowering. FT belongs to the PEBP gene family, which includes five other member *TWIN SISTER OF FT* (*TSF*), *TERMINAL FLOWER 1* (*TFL1*), *A. thaliana CENTRORADIALIS* homolog, *MOTHER OF FT AND TFL1* (*MFT*), and *BROTHER OF FT AND TFL1* (*BFT*)^19–23^. *TFL1* acts as a competitor of *FT* by competitive binding to *FD*^24^, which in turn activates the floral meristem identity gene *APETALA1* (*AP1*).

In legumes, the *FT* genes belong to three distinct subclades, *FTa*, *FTb* and *FTc*, each of which comprises several genes with distinct expression patterns in pea^25^. The two key *FT* genes in leaves, *FTa1* and *FTb2*, generate distinct mobile signals that influence flowering at the apex. *FTb2* showed the highest induction under LD conditions in leaves, whereas *FTa1* and *FTc* showed expression in apical buds. Hecht^25^ proposed a model for the role and interactions of the pea *FT* genes, with *FTa1* acting as a photoperiod-independent monitor of environmental variables that trigger the flowering, whereas *FTb2* functions as the primary signal in leaves for promoting flowering in LD conditions.

Two legume crops stand out in flowering time studies, pea and soybean, with more than 20 loci identified in pea and at least 10 in soybean^14^. The majority of these loci may have been detected as QTLs in related legume crops but the correspondence is difficult to assess due to a lack of common markers and/or sequence information. For example, in chickpea or lentil, major QTLs corresponding to flowering time have been reported^26–31^, but only in a few cases^32^, the orthology with other crops or model species has been assessed. In case of faba bean, just a few QTL analyses for flowering time have been reported^33–36^ but no information on the underlying genes and possible orthology, is currently available.

The aim of this study was to exploit translational genomics from model species or related legume crops to identify QTLs linked to flowering time in faba bean. For the first time, a set of genes relevant for flowering control in *A. thaliana*, *M. truncatula*, *P. sativum* and *C. arietinum* were assayed for polymorphisms and further mapped in a segregating faba bean RIL population. The objective was to saturate the map with candidate genes and to identify the QTLs that show a conserved location across different species. The results from this study will advance the understanding of the genetic basis of flowering time control and identify candidate genes and genomic regions of use for targeted molecular breeding of the trait in faba bean.

## Results

### Phenotypic evaluation

Three different flowering time traits were evaluated: DF1 (days from sowing to appearance of the first flower); DF50 (days until 50% of plants had a visible open flowers); and FL (flowering length, days between the start and the end of the flowering period). All three traits showed a continuous distribution, suggesting that they are controlled by multiple genes in this population (data not shown).

Mean phenotypic values and basic descriptive statistics of the traits are shown in Table 1. The two parental lines showed clear differences, DF1 and DF50 were shorter in Vf27 compared to Vf6, whereas FL was longer in 2007/08. The range of values for the three flowering traits in the RILs showed similar results across the years. DF1_2011/12 showed a higher mean value than in the previous years (DF1_2006/07 and DF1_2007/08) while the mean values in Lebanon for DF50 (DF50_2009/10 and DF50_2010/11) were lower than those recorded in Spain (DF50_2011/12).

**Table 1:** Phenotypic values (mean ± SE) of days to first flower (DF1), days to 50% of flowering (DF50) and flowering length (FL) for parental and RILs from the cross Vf6 × Vf27 in each location and agronomic season.

Trait	Location	Vf6	Vf27	Range (min–max)	Mean (± SE)
DF1_2006/07	Córdoba, Spain	108	98	87–131	105.8 ± 0.88
DF1_2007/08	Córdoba, Spain	102	82	79–125	100.6 ± 0.96
DF1_2011/12	Córdoba, Spain	–	–	101–137	120.6 ± 0.91
DF50_2009/10	Terbol, Lebanon	123	105	101–135	115.5 ± 1.01
DF50_2010/11	Terbol, Lebanon	131	115	105–133	120.6 ± 0.64
DF50_2011/12	Córdoba, Spain	–	–	104–144	130 ± 0.94
FL_2006/07	Córdoba, Spain	–	57	35–70	53.07 ± 0.84
FL_2007/08	Córdoba, Spain	48	57	20–66	43.79 ± 0.92

The correlation analysis between the phenological traits DF1, DF50 and FL and years is summarized in Table 2. All correlations were significant (*p* value < 0.01) and the three traits displayed a high degree of intercorrelation. DF1 and FL in 2006/07 and 2007/08 showed high positive correlation (0.83 and 0.74, respectively), while strong negative correlations were observed between DF1 and FL in both seasons. This is due to trait compensation effects since the earlier the plants flower, the longer the flowering period lasts. A very high correlation value was also found between DF1_2011/12 and DF50_2011/12 (0.94) and between the two evaluations performed in Terbol, Lebanon (DF50_2009/10 and DF50_2010/11).

**Table 2:** Flowering time traits correlation values.

	DF1_2006/07	DF1_2007/08	DF1_2011/12	DF50_2009/10	DF50_2010/11	DF50_2011/12	FL_2006/07	FL_2007/08
DF1_2006/07	1.00							
DF1_2007/08	0.83	1.00						
DF1_2011/12	0.55	0.50	1.00					
DF50_2009/10	0.67	0.67	0.64	1.00				
DF50_2010/11	0.45	0.49	0.51	0.86	1.00			
DF50_2011/12	0.63	0.55	0.94	0.66	0.52	1.00		
FL_2006/07	− 0.93	− 0.78	− 0.51	− 0.61	− 0.57	− 0.58	1.00	
FL_2007/08	− 0.76	− 0.96	− 0.49	− 0.60	− 0.43	− 0.52	0.74	1.00

All the correlations showed a significant *p* value (< 0.01).

The ANOVA results for the genotype by environment (G × E) analyses of each phenological trait are shown in Supplementary Table 1. The analysis revealed significant effects for genotypes (RILs), environment (years) and G × E interactions, identifying G × E interactions as an important factor in flowering time variation across genotypes and years.

### Identification of flowering-related genes and primer design

We selected 31 flowering-related genes from *A. thaliana* (20 genes) and *chickpea* (11 genes) to study their implication in the control of flowering time in faba bean. Protein sequences were used in a BLASTp search to identify orthologous sequences in three legume species close to faba bean, *C. arietinum*, *M. truncatula* and *P. sativum*. Positive blast matches are summarized in Supplementary Table 2. Interestingly, the BLASTp search with *A. thaliana* CRY2 failed to return a positive match in *C. arietinum* and *M. truncatula*.

The sequences of these orthologues were used for designing primers (see materials and methods for details). Information on the primer sequences, size of the amplified genomic fragments and annealing temperatures for each candidate gene are shown in Supplementary Table 3. When applied to faba bean accessions, 28 out of 31 markers yielded amplification products. However, markers Vf_PM3, Vf_SPL1 and Vf_TICb from *C. arietinum* failed to amplify in faba bean.

The amplified sequences from the two parental lines were aligned to search for polymorphisms. Markers Vf_COP1 and Vf_FT produced DNA fragments of different length, which could be directly discriminated by agarose gel electrophoresis. These amplified length polymorphisms (ALPs) are derived from insertions or deletions yielding a different banding pattern. Markers Vf_PIF3 and Vf_GI did not show polymorphisms between the parental lines, while the remaining 24 markers (16 from *A. thaliana* and eight from *C. arietinum*) all revealed SNP polymorphisms. In 18 of these markers, the SNP was associated with a restriction site and thus these markers were transformed into CAPs. For the remaining six markers internal primers were designed. If the internal primer discrimination failed (Vf_TT8, Vf_FKF1 and Vf_PHYA), the SNPs were genotyped using the MassArray iPLEX (Sequenom) platform (http://www.cegen.org). Only Vf_ELF4 could not be genotyped using any of the genotyping techniques described.

### Genetic mapping

**Figure 2:**
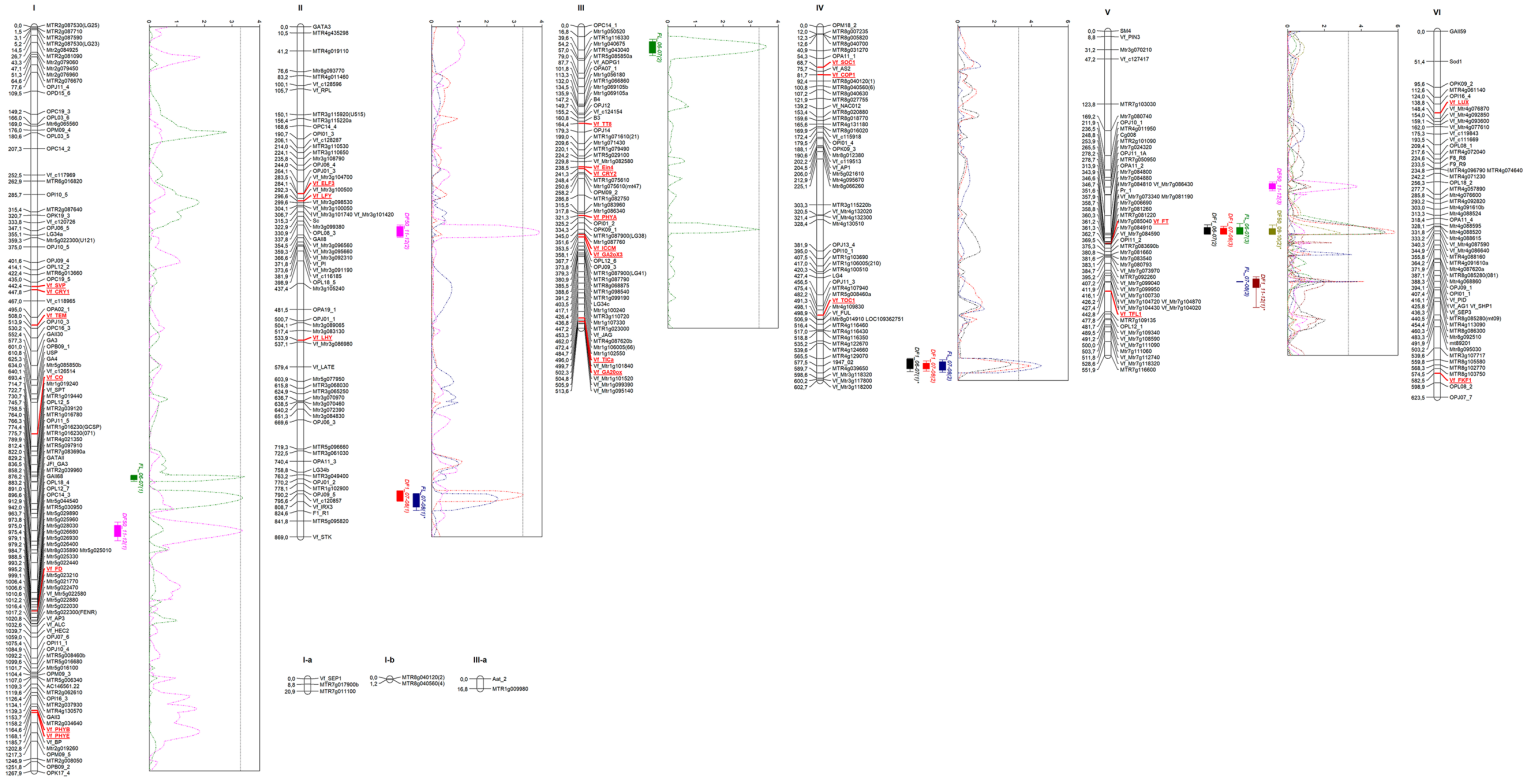
Linkage map and QTLs for flowering time traits detected in the Vf6 × Vf27 RIL population. QTL locations are represented with bars (2-LOD interval) and boxes (1-LOD interval). Candidate gene markers included in the map are highlighted in red. DF1: days to first flower, DF50: days to 50% of flowering, FL: flowering length. No significant QTLs were identified with *.

The genotypic data obtained with the flowering time candidate markers were combined with the data set previously reported in this RIL population^33,37^. Information on the map positions is shown in Table 3. All the new markers fit the expected 1:1 Mendelian ratio except for Vf_FT which showed a distorted segregation skewed towards the Vf6 allele (Table 3). The linkage map, spanning 4.184 ‬cM, consists of six main linkage groups and five smaller arrays (Fig. 2). The existence of common markers with previous consensus maps^34,38^ allowed us to assign all the LGs to the corresponding faba bean chromosomes (chr.). All candidate-gene markers were included in the new genetic map, with numbers ranging from one in LGVI to six in LGI and LGIII.

**Table 3:** Genetic segregation, chi-square test and location of the flowering time gene markers in faba bean (Vf) and related legume species: Ca, *Cicer arietinum*; Mt, *Medicago truncatula* and Ps, *Pisum sativum*).

Marker	X^2^ (1:1)	*P*	Vf_Chr	Ca_Chr	Mt_Chr	Ps_LG
Vf_EIN4	0.59	0.442	**3**	**4**	**1**	Scaffold
Vf_GA20ox	2.086	0.149	**3**	**4**	**1**	**2**
Vf_GA2ox3	2.922	0.087	**3**	**4**	**1**	**2**
Vf_ICCM	0.011	0.916	**3**	**4**	**1**	**2**
Vf_TICa	3.658	0.056	**3**	**4**	**1**	**2**
Vf_TT8	0.385	0.535	**3**	**4**	**1**	**2**
Vf_LHY	0	1	2	3	7	5
Vf_CO	1.316	0.251	1	4	7	5
Vf_COP1	0.91	0.34	4	2	5	1
Vf_CRY1	0.976	0.323	**1**	**2**	**5**	**1**
Vf_CRY2	0.05	0.823	**3**	–	–	**2**
Vf_ELF3	0.047	0.829	**2**	**5**	**3**	**3**
Vf_FD	0.419	0.518	**1**	**8**	**5**	**1**
Vf_FKF1	0.235	0.628	**6**	**6**	8	**6**
Vf_FT	26.385	0*	**5**	**3**	6	**6**
Vf_TFL1	0.400	0.527	**5**	**3**	**7**	**5**
Vf_LFY	1.754	0.185	**2**	**5**	**3**	**3**
Vf_LUX	1.136	0.286	**6**	**6**	**4**	**7**
Vf_PHYA	1.704	0.192	**3**	**4**	**1**	**2**
Vf_PHYB	0.117	0.732	**1**	1	**2**	**6**
Vf_PHYE	0.59	0.442	**1**	1	**2**	**6**
Vf_SOC1	0.048	0.827	**4**	3	**8**	–
Vf_SVP	1.988	0.159	**1**	Scaffold	**5**	**1**
Vf_TEM	0.049	0.825	**1**	Scaffold	**5**	**1**
Vf_TOC1	0.762	0.383	**4**	**7**	4	**4**

Bold values highlight syntenic chromosomes (Chr)/and linkage groups (LGs).

Lack of fit to the expected 1:1 segregation is indicated by an asterisk.

We next checked whether the marker genes share syntenic positions in *V. faba* and the closely related species *M. truncatula*, *P. sativum* and *C. arietinum* (Table 3). The level of synteny was quite high (69%), with 18 out of 25 gene markers conserved in at least one other species. Twelve *V. faba* genes were syntenic with *C. arietinum*, 15 with *M. truncatula* and 16 with *P. sativum*. Finally, 12 markers (Vf_GA20ox, Vf_GA2ox3, Vf_ICCM0293, Vf_TICa, Vf_TT8, Vf_CRY1, Vf_ELF3, Vf_FD, Vf_TFL1, Vf_LFY, Vf_LUX and Vf_PHYA) showed conserved synteny in all four species tested, preserving co-localization of these genes in their homologous chromosomes.

### QTL analysis

We identified 12 significant QTLs (LOD threshold > 3.3) related with flowering time in chr. I, II, III, IV and V (Table 4, Fig. 2). Except for DF1_07-08(1), DF1_07-08(2), DF50_11-12(1) and FL_06-07(3) the additive effects of the associated markers were positive, indicating that most early flowering alleles originate from the Vf27 parental line.

**Table 4:** QTLs for flowering time traits identified in the Vf6 × Vf27 RIL population.

Trait	Peak	Chr	Flanking markers	LOD	Additive effects	R^2^
FL_06-07(1)	766.313	I	OPJ11_5	3.46	2.27494	7.4
DF50_11-12(1)	858.208	I	Mtr2g039960	3.39	− 2.91921	9.1
DF50_11-12(2)	349.829	II	GAII8/Vf_Mtr3g096560	3.91	3.90166	17.9
DF1_07-08(1)	796.562	II	Vf_c120857	3.31	− 3.06379	10.7
FL_07-08(1)*	805.562	II	Vf_IRX3	2.39	2.4726	7.6
FL_06-07(2)	33.823	III	Mtr1g116330	3.57	2.86524	11.6
DF1_06-07(1)*	577.528	IV	1947_02	2.75	− 2.10807	5.3
DF1_07-08(2)	577.528	IV	1947_02	3.98	− 3.16022	11
FL_07-08(2)	577.528	IV	1947_02	4.54	3.17776	12.6
DF50_11-12(3)	265.522	V	Mtr7g024320	3.72	3.36068	13.2
DF1_06-07(2)	343.911	V	Mtr7g084800	5.26	3.85206	11.1
DF1_07-08(3)	343.911	V	Mtr7g084800	5.76	4.90158	17.4
DF50_09-10(2)*	343.911	V	Mtr7g084800	2.86	4.53734	12.8
FL_06-07(3)	343.911	V	Mtr7g084800	5.19	− 3.41023	12.2
DF1_11-12(1)*	426.159	V	Vf_Mtr7g104720/Vf_Mtr7g104870	2.57	− 3.22325	10.6
FL_07-08(3)	426.167	V	Vf_Mtr7g104870	3.80	3.29528	11.1

*No significant QTL.

We identified 4 significant QTLs for DF1. Two of these, DF1_06-07(2) and DF_07-08(3), with a LOD peak > 5, explaining 11.1 and 17.4% of the phenotypic variance, respectively, colocalized in chr. V associated with the *Medicago truncatula* (Mtr) marker Mtr7g084800. In addition, DF1_07-08(3) showed a second significant peak that colocalized with FL_07-08(3) and explained 11.1% of the variation. The other two QTLs were located in chr. II (DF1_07-08(1)) and IV (DF1_07-08(2)) and explained 10.7 and 11% of the trait variation.

For DF50 scored in 2011/12, three significant QTLs were found in chr. I, II and V. The largest main effect QTL was DF50_11-12(2), which explained 17.9% of the phenotypic variance and is located in chr. II flanked by GAII8 and Vf_Mtr3g096560.

Finally, 5 significant QTLs for FL were detected in chr. I, III, IV and V. Two of these colocalized with other DF1 QTLs in chr. IV (FL_07-08(2)) and chr. V (FL_06-07(3)) (Fig. 2). Moreover, two additional QTLs for FL_2006/07 were detected in chr. I (with a second peak between Mtr4g021350 and Mtr5g097910) and chr. III (with a second peak next to Mtr1g087900(LG38)), explaining 7.4 and 11.6% of phenotypic variation, respectively.

Four medium-sized QTLs failed to reach the defined threshold: FL_07-08(1) in chr. II, DF1_06-07(1) in chr. IV and DF1_11-12(1) and DF50_09-10(2) in chr. V. Nevertheless, these QTLs are displayed in Fig. 2 and Table 4 since they co-localized with other significant QTLs. However, none of the 25 flowering genes included in the map appear to be plausible candidates for the observed QTLs, and only FT and TFL1 (in chr. V) were located close or within the confidence interval of a significant QTL.

In summary, chromosome V accumulates most of the consistent and stable flowering time QTLs detected across different locations and years. These QTLs were distributed in two regions. The first including QTLs for DF1, DF50 and FL is located close to the marker Mtr7g084800, while the second including QTLs for DF1 and FL is close to Vf_Mtr7g104870. Additional relevant genomic regions were identified in chr. II and IV, where DF1 and FL QTLs colocalized in the distal part of both chromosomes and explained between 10.7–17.9% and 11–12.6% of the phenotypic variation, respectively.

To identify the orthologous genes flanking the QTLs, we performed a BLASTp search against the *Arabidopsis* protein database in the NCBI platform. These results are shown in Supplementary Table 4.

## Discussion

Flowering time is one of the most important adaptive traits in plants^39^ and its regulation crucially affects crop yield. As many other temperate legume crops, faba bean synchronizes flowering to match the seasons by monitoring cues such as temperature and photoperiod. Thus, exposure to winter cold (vernalization) results in the competence to flower during the following spring in response to increasing day length signals.

Both the vernalization pathway and the photoperiod pathway have been characterized in detail for the model plant *A. thaliana*, where more than 100 genes contributing to the flowering time control have been identified^40^. Most of the key genes and gene families of the *Arabidopsis* flowering time pathway appear to be conserved in legumes, with a few of them having undergone duplications (e.g. *CRY2*) or loss (e.g. *PHYC*)^14^. Thus, there is significant potential for translating the knowledge on flowering control from the model or related legume species to faba bean.

Here we conducted a comprehensive analysis of the genetic determination of flowering time in faba bean by systematically evaluating three related traits in a segregating RIL population across different locations and years using a combination of comparative genomics, candidate gene mapping and QTL analysis. We selected a set of 31 genes controlling flowering time in *Arabidopsis* and chickpea to fine map faba bean target regions and identify genes co-localizing with previously identified QTLs, and were able to genotype and include 25 of them in the faba bean linkage map. Most of these markers showed a conserved synteny with the related legume crops *Medicago*, *Pisum* and *Cicer* (Table 3), although none of them co-localized with previously reported faba bean QTLs. Importantly, only *FT* gene mapped close to the most prominent and consistent QTL region (see below).

The analysis identified 12 significant QTLs for flowering time. The most important region in chr. V, contained five QTLs for different flowering traits that were stable and consistently detected across different years. The co-localization of two putative QTLs, DF1_11-12(1) and DF50_09-10(2), which did not reach the established significance level, further corroborates the importance of this genomic region flowering time control.

This region on chr. V was first identified by Cruz-Izquierdo^33^, with a major QTL near markers Pis_GEN_6_3_1 (Mtr7g084800) and AnMtS37 (Mtr7g081220). Subsequent studies in faba bean^35,36^, showed a relation of days to flowering with the SNP marker Vf_Mt7g084010, which mapped close to Mtr7g084800. Our present results confirm that Mtr7g084800 is the marker mapping closest to the most relevant QTL peak (Table 4, Fig. 2). Mtr7g084800 corresponds to a photosynthetic glyceraldehyde-3-phosphate dehydrogenase (GAPDH), which participates in the Calvin cycle, is transcriptionally regulated by light^41^ and highly expressed in leaves and floral stalks^42^.

Studies in temperate legume species such as *M. truncatula*, chickpea, narrow-leafed lupin, alfalfa and L*otus japonicus* confirmed the conservation of this major flowering time QTL in a region syntenic with a section of *Medicago* chr. 7, containing a tandem array of *FTa* and *FTC* genes (reviewed by Weller and Ortega^14^). As mentioned above, we found that *FT* was the only candidate gene mapping close to this QTL region, where marker Mtr7g084800 is surrounded by a cluster of *FT* flowering time loci (Supplementary Table 5). We attempted to design primers pairs corresponding to the different *FT* candidate genes present in the *M. truncatula* QTL interval^43^ but the high sequence conservation prevented further amplification. Using the available *M. truncatula*, *P. sativum* and *V. faba FT* sequences, we were able to design primer pairs for Mtr7g085040 (*FTC*) as well as for a second *V. faba FT*, although linkage analysis mapped both markers to the same position confirming the high sequence conservation. Because none of the new FT markers genotyped was directly linked to the QTL found in chr. V, the identity of the responsible gene(s) remains to be determined.

Also, in chr. V, marker Vf_Mtr7g104870 was associated with QTL FL_07-08(3) and DF1_11-12(1), although the latter did not reach the significance level. Vf_Mtr7g104870 shows homology with the *A. thaliana* AT4G18590 gene encoding one of the three subunits of DNA Replication Protein A (RPA). RPA is a protein complex with a critical function in DNA replication, repair, recombination and epigenetic maintenance. These proteins play important roles in epigenetic gene silencing and in the regulation of the meristem development in *A. thaliana*^44^. Interestingly, vernalization in *Arabidopsis* involves downregulation of *FLC*, primary floral repressor^45,46^. Bastow^47^ showed that vernalization causes histone methylation in the *FLC* gene, thereby preventing the transition to the reproductive state.

Further, we detected QTLs in chr. I, II, III and IV. The QTL DF50_11-12(1) in chr. I was associated with marker Mtr2g039960, which corresponds to eukaryotic translation initiation factor 4A1 (*EIF4A1*) according to a BLASTp search against the *A. thaliana* genome database (Supplementary Table 3). *EIF4A1* was previously shown to modulate the light/dark cycle in mature leaves^48^. Interestingly, *A. thaliana* mutants in *EIF4A1* showed a pronounced late-flowering phenotype and significantly altered expression of *FLC* and *SOC1*, a positive and negative regulator of *FT* genes, respectively^49^. Mutation in another *EIF4* gene (*EIF4G*) of *A. thaliana* resulted in a late flowering phenotype^50^. In maize, *EIF4A1* was also described as a candidate gene for flowering time^51^.

In chr. II we detected QTL DF1_07-08(1), which colocalizes with FL_07-08(1), although it did not reach the significance level. Both are associated with markers Vf_c120857 and Vf_IRX3. Vf_c120857 (Mtr3g449590) shows homology with AT4G23820, a pectin lyase-like superfamily protein, which is apparently unlinked to flowering. A second QTL in chr. II, DF50_11-12(2), is flanked by markers GAII8 and Vf_Mtr3g096560. While GAII8 is a microsatellite, Vf_Mtr3g096560 corresponds to *A. thaliana*, DNA polymerase delta (***δ***) subunit 1 (AT5G63960), which is part of a complex of three DNA polymerases. DNA polymerase ***α*** (Pol***α***) initiates DNA strand synthesis while Polε and Pol***δ*** perform the synthesis in the leading and lagging strands, respectively. In *A. thaliana*, Pol***δ*** was reported to regulate flowering via epigenetic marks^52^. According to the proposed model Pol***δ*** is delayed at high temperatures, triggering a DNA replication stress response which results in methylation of a H3K4me3 histone in the *SEPALLATA3* (*SEP3*) locus. This epigenetic mark promotes transcription *SEP3*, which participates in a feedback loop with *FT* genes, thus linking Polδ activity to the establishment of transcriptional epigenetic marks affecting flowering. These findings are in line with the fact that in *Arabidopsis esd7* and *abo4-1* mutants, discrete regions at the *FLOWERING LOCUS T* (*FT*) are enriched in H3K4me3, correlating with higher *FT* and lower *FLC* expression levels^53^.

The QTL FL_06-07(2) in chr. III was associated with Mtr1g116330, a PRLI-interacting factor specifically expressed during flower development in *Acacia mangium*^54^. In rice, genetic analysis and fine mapping of a set of complementary genes controlling late heading (flowering time) also identified a PRLI-interacting factor as a putative candidate for LH1^55^.

Finally, consistent QTLs for DF1 and FL co-localized in chr. IV were associated with the 1947_02 marker corresponding to the *M. truncatula* Mtr4g132540 gene, a homolog of the jumonji domain-containing protein JmjC in *A. thaliana* (Supplementary Table 3). JmjC proteins regulate epigenetic processes involved in plant growth, development and disease resistance^56–58^. In *A. thaliana* several JmjC members have been characterized. They play a critical role in flowering time control via chromatin remodeling involving modifications of histones that either promote or repress gene expression. Members of the two known families of histone demethylases in eukaryotes, lysine‐specific demethylase 1 (LSD1) and Jumonji C‐terminal domains are involved in regulating both defense and developmental (flowering time) processes^59,60^, as now is also suggested in faba bean.

The QTL analysis identified *EIF4A1* in chr. I and PRLI-interacting factor in chr. III, as well as three candidate genes involved in flowering time regulation through histone modifications, *RPA* in chr. V, DNA polymerase delta (δ) in chr. II and JmjC in chr. IV. This result highlights the important role of epigenetic modifications in faba bean flowering control. Based on this finding, we propose that conserved epigenetic pathways in plants can be exploited as a novel source to identify markers for flowering control and stress tolerance^61^.

The QTLs and candidate genes identified here provide a robust framework for further genetic studies aimed at elucidating the mechanisms controlling flowering time. The ongoing faba bean genome sequence assembly together with the development of dense, gene-based genetic maps will further facilitate fine mapping of relevant QTL regions. These QTLs need to be transferred to effective markers for further use in the breeding programs. Therefore, additional flowering time studies involving new faba bean genetic backgrounds and expression analyses will validate the usefulness of the identified candidates in flowering control. The predicted outcomes will provide targets for an effective selection of flowering traits in faba bean breeding programs.

## Material and methods

### Plant material

A recombinant inbred line (RIL) population of 124 individuals derived from the faba bean cross Vf6 × Vf27 was used in this work. Vf6 is an *equina* medium-seeded field bean with beige seed coat and late flowering time while Vf27 is a black and small-seeded *paucijuga* primitive form with earlier date of flowering, with a phenotype similar to the hypothetical wild progenitor^62^. This RIL population was developed at IFAPA and well-studied in previous mapping and QTL research^63–65^.

### Phenotypic evaluation

Evaluations were conducted at IFAPA-station in Alameda del Obispo, Cordoba in Spain (37.51° N, 4.48° W, 94 m) during 2006/2007, 2007/2008 and 2011/2012 seasons) and at ICARDA, Terbol Research Station, Bekaa Valley in Lebanon (35.98° N, 33.88° E, 890 m) during 2009/2010 and 2010/2011 winter cropping seasons. The mean annual temperature in Córdoba is 24.6 °C and the annual rainfall is 536 mm, mostly concentrated from December to February. In Terbol, the mean annual temperature is 15.4 °C and the annual rainfall of 559 mm. A randomized complete block design with two replicates per genotype was used in both environments. The experimental unit consisted of 10 plants including RILs and parental lines. Sowing dates were during the first half of November in the five growing seasons.

Flowering traits were recorded using different methods: DF1, scored as the number of days from the sowing until the appearance of the first flower; DF50, recorded as the number of days until the 50% of the plants had visible open flowers and FL (flowering length), scored as the difference of days between the start and the end of flowering period (when no flowers were present in the plants). DF1 was evaluated in 2006/07, 2007/08 and 2011/12 (only in Spain); DF50 was evaluated in 2009/10, 2010/11 and 2011/12 (in Spain and Lebanon) and FL was evaluated in 2006/07 and 2007/08 (only in Spain). Evaluations from 2006/07 to 2007/08 were previously reported by Cruz-Izquierdo^33^.

Global means, ranges, standard error (SE), histograms of frequency and statistical analyses were carried using the R software version 4.02^66^.

### Flowering-related genes and primer design

To develop molecular markers associated with flowering time in faba beans, we used a candidate gene-based strategy^67^. Thirty-one flowering-related genes described in other species (mostly in chickpea and *A. thaliana*) were selected (Supplementary Table 2). Then using the BLAST tool, homologous sequences were identified in *Medicago truncatula* and *Pisum sativum.* Positive results were used as well to search for homologous sequences in faba bean transcriptome databases^68^. The obtained sequences were aligned using the Geneious software suite (v. 7.1.9; Biomatters, Auckland, New Zealand) to identify conserved domains and design the primer pairs. When possible, primers were designed on faba bean sequences, otherwise, we used the sequence of related species.

A set of exonic primers flanking the intronic sequences with a size of 20–25 pb, GC contents of 45–60% and Tm of 60 ± 1 °C were designed to amplify fragments in a 300–1200 bp range. The markers developed in this work were named as the corresponding candidate gene preceded by Vf (Supplementary Table 3).

### PCR amplification and polymorphism detection

Genomic DNA was isolated from young leaves using a CTAB protocol^69^. PCR amplification consisted in 25 μl of reaction containing 4 ng template DNA, 1 × PCR buffer, 2 mM of MgCl_2_, 0.4 mM of dNTPs, 0.2 μM of each primer and 1 unit of Taq polymerase (Biotools B&M Labs, Madrid, Spain). The amplification profile was an initial denaturation at 94 °C for 5 min, followed by 30 cycles of 45 s at 94 °C, 45 s at 56–60 °C and 45 s at 72 °C, with a final extension step of 8 min at 72 °C. PCR products were separated using 2% agarose gels. Positive amplifications were purified using a standard 3 M sodium acetate (pH 4.5) and ethanol protocol. The purified DNA was used for direct sequencing (STABVIDA, Portugal). Finally, the sequences from parental and contrasted RILs for flowering time were aligned to detect SNPs (Single Nucleotide Polymorphisms). Those SNPs with recognition sites for restriction enzyme digestion between lines were converted into CAPS makers (Cleaved Amplified Polymorphism Sequence) to genotype the whole RIL population. Restriction digestions were performed following the supplier’s instructions and visualized in 2% agarose gels. For SNPs lacking restriction enzymes in the polymorphic site, internal primers were designed using the technique Tetra-Primer ARMS–PCR described by Medrano and Oliveira^70^. When the internal primers did not yield amplification products or a clearly scorable segregation, the genotyping was carried out at CEGEN-PRB3-ISCIII (http://www.cegen.org) using the MassArray iPLEX (Sequenom) SNP genotyping platform from the Spanish National Genotyping Center facility of the University of Santiago de Compostela.

### Genetic mapping and QTL analysis

The preceding maps developed in the Vf6 × Vf27 RIL population include approximately 425 RAPDs, ESTs and SNP markers^33,37^. The new polymorphic flowering time markers were genotyped in this RIL population and incorporated into the previous dataset^33,37^ to develop a new linkage map. Chi-squared tests to determine goodness of fit to the expected segregation ratio of 1:1 were carried out for all markers segregating in the RIL population. Linkage analysis was performed using JoinMap v4.0^71^ with maximum likelihood option. Markers were grouped at a minimum LOD score of 3 and a maximum recombination fraction of 0.25 as general linkage criteria to establish linkage groups. Kosambi’s function was applied to convert the recombination frequencies into genetic distances^72^.

QTL analysis was conducted by MapQTL v5.0 software^73^. First, the nonparametric Kruskal–Wallis test was performed to estimate the marker significance level and the marker-phenotype association. Then, the interval mapping^74,75^ was applied to identify putative QTLs in each linkage group (LG). Markers significant at *p* = 0.01 were used as cofactors in the multiple QTL analysis (MQM)^76–78^. QTL significance (*p* value) was determined by permutation analysis using 1000 replicates^79^, as implemented in MapQTL 5.0. Only QTLs with a LOD higher than the p-value were declared as significant. MapChart software^80^ was used to represent the QTLs confidence interval. The support intervals were defined as LOD-1 and LOD-2 around the maximum LOD of QTL. If QTLs for the same trait detected in different environments had overlapping 2-LOD support intervals, they were considered to be the same QTL and also been designated as consistent QTLs.

### Ethical approval

Faba bean is a common crop extensively cultivated in the world. The authors declare that the experimental research work conducted in this study was carried out in accordance with relevant guidelines and regulations.

## Supplementary Information

Supplementary Information.

## Data Availability

The datasets generated and analyzed during the current study are available from the corresponding author on request.
